# A Novel Artificial Intelligence-Enabled Method for Electronic Nose Design Based on Olfactometry Data

**DOI:** 10.3390/s26072150

**Published:** 2026-03-31

**Authors:** Gizem Teker, Taner Yonar, Enes Yiğit

**Affiliations:** 1 Faculty of Electrical and Electronics Engineering, Bursa Uludag University, Bursa 16059, Türkiye; 2 Envora R&D Engineering Inc., Ulutek Technology Development Zone, Bursa 16059, Türkiye; 3 Faculty of Environmental Engineering, Bursa Uludag University, Bursa 16059, Türkiye

**Keywords:** electronic nose, olfactometry, n-butanol, TS EN 13725, machine learning, odor quantification, metal oxide sensors

## Abstract

**Highlights:**

**What are the main findings?**

- An innovative electronic nose system was successfully developed and validated against the TS EN 13725 dynamic olfactometry standard using n-butanol as a reference, enabling the digitalization of odor analysis traditionally dependent on human perception.
- Among the tested machine learning models, Support Vector Regression (SVR) demonstrated superior performance with a Test R^2^ of 0.987 and a low Test MAPE of 11.09%, effectively quantifying odor concentrations.

**What are the implications of the main findings?**

- The proposed methodology provides a standardized and objective instrumental alternative to highly variable and error-prone human perception-based odor measurements in environmental monitoring.
- The high generalization capability of the SVR model on independent datasets validates the system’s potential for reliable and low-cost odor quantification in industrial and environmental applications, paving the way for real-time and dynamic field-deployable odor monitoring technologies.

**Abstract:**

Electronic nose systems are advanced technological tools that enable the objective evaluation of odors through sensor arrays mimicking the human olfactory mechanism and sophisticated data processing algorithms. These systems facilitate rapid, reproducible, and standardized measurement of chemical components in applications such as food safety, environmental monitoring, medical diagnostics, and industrial quality control. In this study, measurements obtained from electronic nose sensors were compared with olfactometry panelist assessments using n-butanol as a reference substance in accordance with the TS EN 13725 standard. Furthermore, machine learning algorithms, including Partial Least Squares (PLS), Support Vector Regression (SVR), and Gaussian Process Regression (GPR), were applied to model the sensor data and evaluate their predictive accuracy. The results demonstrated the reliability and applicability of the electronic nose system, achieving training mean absolute percentage error (MAPE) values of 6.53% for PLS, 10.89% for SVR, and 0.15% for GPR. This study presents an innovative approach that systematically assesses the performance of electronic nose technology using a standardized reference odor and highlights the effectiveness of the modeling approach.

## 1. Introduction

Electronic nose (E-nose) systems are devices that enable the objective analysis of odors through sensor arrays mimicking the human olfactory mechanism and data processing algorithms [1]. Compared to traditional laboratory methods, they offer rapid, portable, and reproducible measurements, making them widely used in various fields such as robotics [2], food engineering [3,4,5,6], environmental monitoring [7,8], and disease diagnostics [9,10].

In the domain of environmental engineering and air quality monitoring, odor measurement presents a unique and critical challenge. While the concentrations of most other air pollutants are determined through fully automated sensors and analytical instruments, the assessment of odor perception remains the single parameter in environmental quality management that fundamentally relies on the human sensory mechanism (human nose). Dynamic olfactometry, the legally binding method for determining odor concentration, is therefore inherently dependent on the subjective responses of a trained human panel. This dependency introduces significant limitations regarding the cost, duration, and inter-laboratory reproducibility of the measurement results, thereby underscoring the necessity for objective instrumental alternatives.

An electronic nose consists of sensors capable of detecting different gases, which mimic the odor receptors in the human nose [1]. The sensor data allow each odor to produce a characteristic pattern that can be considered a unique “fingerprint” [11]. These patterns are analyzed using machine learning algorithms to identify specific odors and accurately classify similar odors in the future [1]. High accuracy rates for E-nose systems were demonstrated in various applications, such as 90.7% accuracy in evaluating fish freshness using a decision tree algorithm [6] and nearly 100% distinguishability in beverage analysis using metal oxide sensors [5]. Artificial neural networks (ANN) and k-nearest neighbor (k-NN) algorithms were also successfully applied for classifying n-butanol concentrations [12]. Borowik et al. [13] showed that a custom-built E-nose with a Figaro TGS gas sensor array could discriminate between pristine and hydraulic oil-contaminated soil with up to 97% accuracy using sensor heater modulation. Poeta et al. [14] demonstrated that MOX sensor-based E-noses, combined with GC-MS analysis, could reliably distinguish authentic PDO (geographically certified) extra virgin olive oil from adulterated samples, providing a rapid and cost-effective tool for food authenticity assessment. In agriculture, Martínez et al. [15] reported 100% differentiation between healthy and *Monilinia laxa*-infected plums, confirming the potential of E-noses as non-destructive, real-time tools for postharvest quality monitoring. These studies illustrate the qualitative characterization capabilities of E-noses, in contrast to the quantitative odor concentration measurements targeted in the present work. N-butanol is designated by the TS EN 13725 standard as the reference substance for odor measurement [16]. Its strong and characteristic odor is essential for odor calibration, ensuring that electronic nose measurements can be compared with olfactometry-based human perception tests.

Beyond industrial and environmental uses, specialized devices like the Cyranose 320 (Sensigent, Baldwin Park, CA, USA), proved that electronic noses could even handle complex medical tasks. By using a compact array of 32 sensors, lung damage in post-COVID patients was successfully identified by this specific product through breath analysis. The system works by capturing a unique ‘breath-print’ and processing it with smart algorithms, achieving high accuracy in distinguishing healthy individuals from those with respiratory issues. This example highlights how portable E-nose technology can replace slow, expensive laboratory tests with fast and objective digital measurements [17].

Recent advancements in electronic nose technology have demonstrated significant potential in quantitative odor analysis and VOC profiling across various fields. For instance, recent studies have successfully employed sensor arrays and advanced deep learning models for food quality assessment, such as analyzing milk using biosensors [18] and roasted walnuts [19], as well as for agricultural applications like detecting volatile pheromones of invasive pests [20]. While these studies excel in specific chemical concentration estimations, the quantitative assessment of hazardous and toxic gases presents a far more critical challenge. Several E-nose systems have been developed specifically for predicting combustible and toxic gas concentrations to ensure environmental and industrial safety [21,22]. However, state-of-the-art studies emphasize that E-nose systems deployed in real-world hazardous environments still face severe technical limitations. Recent research highlights that these devices struggle with feature distribution shifts caused by sensor drift, unknown background gas interferences, and significant sensitivity variations confounded by environmental factors such as humidity and pressure [23,24].

Crucially, these existing quantitative devices are typically not calibrated using a universally standardized reference substance, such as n-butanol, as mandated by the dynamic olfactometry standard (TS EN 13725). This lack of standard compliance severely restricts their application as a direct and reliable replacement for human expert panels. The ability to completely replace human assessors is profoundly important in toxic, hazardous, and heavily industrialized environments, where exposing trained panelists to noxious emissions poses severe occupational health risks. Therefore, there is an urgent and critical need for standard-compliant, AI-enhanced electronic noses that can overcome existing sensitivity limitations and directly translate raw sensor data into standardized Odor Units (OU/m^3^) safely and reliably.

The electronic nose developed in this study introduces an innovative approach to odor measurement supported by machine learning algorithms. Sensor-based measurements are calibrated using n-butanol as a reference substance according to the TS EN 13725 standard [16] and are compared with olfactometry panelist evaluations. This approach aims to minimize the subjectivity of human perception-based measurements while producing reproducible and objective data.

Importantly, this work directly addresses the critical challenge in environmental engineering: replacing subjective human panel assessments with an AI-driven, sensor-to-odor unit mapping. By training the MOX sensor array with validated olfactometry data, the system bridges human perception and digital measurement, providing a high-precision, low-cost, and repeatable alternative to conventional olfactometry.

The rest of this paper is organized as follows: In Section 2, the proposed methodology, including the electronic nose system design, olfactometry data acquisition, and artificial intelligence-based modeling, is introduced in detail. In Section 3, experimental results along with the performance evaluation of the utilized machine learning models are presented and discussed. Finally, concluding remarks and potential future applications of the proposed system are cited in Section 4.

## 2. Materials and Methods

### 2.1. Odor and Odor Measurement

Odor is defined according to the EN ISO 5492:2009 standard as “a sensation perceived by the olfactory organ during the sniffing of certain volatile substances” [25]. Odor measurement methods include subjective (Olfactometry, Sensory Analyses) and objective (Chemical Analysis, Electronic Noses) approaches. In this study, an odor measurement approach combining Olfactometry and computer-assisted analyses with an E-nose is presented. While Olfactometry relies on the subjective perception of human panelists, the Electronic Nose provides objective signals obtained from gas sensors, offering advantages such as rapid measurements and high reproducibility [1,16].

### 2.2. Electronic Nose

Odors are perceived through the detection of chemical molecules by the nose and their subsequent identification by the brain, with the composition of odor molecules being essential in this process. The discriminative ability of the human nose has inspired the development of devices [26]. For this purpose, electronic nose systems, typically composed of chemical sensor arrays modeled after the human olfactory system, have been developed. These systems can operate faster and with greater sensitivity compared to the human sense of smell. In general, an electronic nose system consists of three main components:

- Sample Gas Delivery Unit: Transfers volatile molecules to the sensor array.
- Detection Unit: Includes a series of sensors that convert chemical signals into electrical signals (Metal oxide sensors (MOS) are preferred for their high sensitivity, wide detection range, and low cost [27]), and an analog-to-digital converter (ADC)
- Computational System: Reads the digital signal and performs analyses.

**Figure 1:**
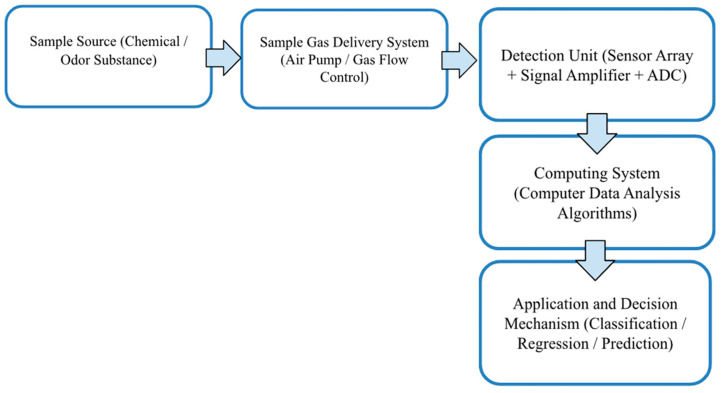
Workflow diagram of the electronic nose.

The developed system is designed to integrate these components, including a plexiglass test chamber for the sensors, pump systems controlling gas flow, a computational module, and a decision support mechanism incorporating machine learning algorithms to enable the precise measurement of different concentrations of n-butanol (Figure 1).

#### 2.2.1. Sensor Characteristics and Setup

Volatile Organic Compounds (VOCs) are organic chemicals that readily evaporate and constitute the basis of virtually all odors perceived by humans. The E-nose system utilizes eight metal oxide gas sensors (MOS), comprising two units of four different types, to record responses to n-butanol. The use of duplicate sensors of each type was intentionally implemented to enhance measurement reliability and robustness. This redundancy allows cross-verification of sensor responses and mitigates the effects of environmental variations, sensor drift, and manufacturing inconsistencies, thereby providing more stable and trustworthy odor data. Furthermore, since environmental odors—including baseline calibrators like n-butanol—are inherently complex mixtures of Volatile Organic Compounds (VOCs), relying on a single sensor is inadequate for accurate quantification. Therefore, the implementation of this specific broad-spectrum sensor array is essential to guarantee collective cross-sensitivity across diverse chemical groups. This multidimensional feature extraction is critical for establishing a robust and consistent response matrix that can be effectively correlated with the unified Odor Unit (OU/m^3^) metric provided by the olfactometry panel.

Sensor characteristics are detailed in Table 1.

In this system, however, the sensors are configured within a voltage divider circuit, enabling the data acquisition unit to record the output voltage directly. Instead of relying on these intermediate resistance conversions, the raw voltage variations are directly fed into the machine learning framework. Because these advanced predictive models inherently map the complex, non-linear relationships between the direct voltage fluctuations and the target odor units (OU/m^3^), calculating the traditional Rs/R_0_ ratio is circumvented, thereby optimizing the computational efficiency of the odor quantification pipeline.

#### 2.2.2. Gas Sample Preparation

**Figure 2:**
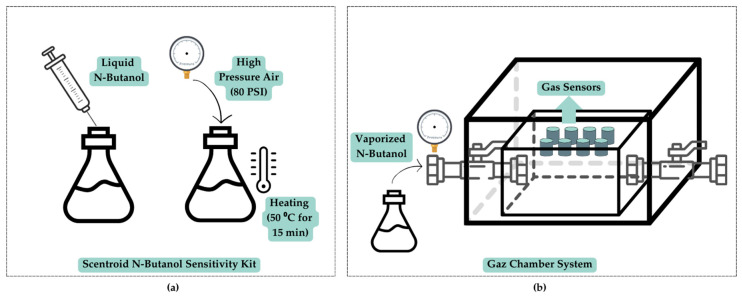
Illustration of system stages: (**a**) gas sample preparation; (**b**) gas chamber system.

To create a standardized dataset, liquid n-butanol was converted into the gas phase using a Scentroid N-Butanol Sensitivity Kit (Scentroid, Toronto, ON, Canada), which includes a Scentroid-branded 10 mL precision gas-tight glass syringe with a PTFE plunger and ±1% volumetric accuracy. High-pressure air (80 PSI) was injected into a stainless-steel canister containing a known volume of liquid n-butanol, and the mixture was heated to 50 °C for 15 min to ensure complete vaporization. Dilutions were prepared either by direct volumetric adjustment using the Scentroid syringe or by incorporating deionized water, a high-purity laboratory water type produced by ion-exchange that contains extremely low ionic content (Millipore, Burlington, VT, USA; Milli-Q Ultra Pure Water System, 18 µΩ·cm). The resulting dilutions were then introduced to the sensor system concurrently with olfactometry experiments (Figure 2a). The sequential stages of the experimental process, from sample preparation to detection, are illustrated in Figure 2.

**Figure 3:**
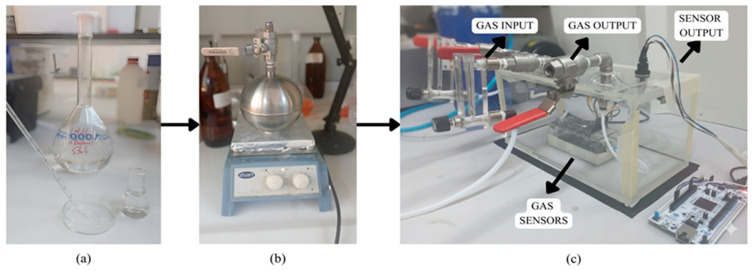
System stages: (**a**) substance dilution stage; (**b**) transition from liquid to gas phase; (**c**) gas introduction to the sensor array.

The final gas detection system developed within the scope of the project is shown in Figure 3c. The system is built on a compact structure to enhance measurement reliability and sensor response accuracy. The sensors are placed in a test chamber made of plexiglass, where only the sensor surfaces come into contact with the gas. The sensor mounting surface is fixed into holes on the side of the enclosure, ensuring that the sensor detection surfaces face directly into the chamber interior, while electronic boards, connection cables, and other circuit elements are positioned outside the enclosure. This configuration ensures that the sensors interact solely with the target gas, eliminating interference from external factors. The sensor enclosure is placed within a larger plexiglass housing. This outer structure provides mechanical stabilization of the system, orderly connection of gas flow lines, and protection from environmental influences during measurement. Gas inlet and outlet lines are connected to the enclosure via metal valves, directing the target gas mixture into the test chamber with the assistance of an air pump. This compact volume allows rapid and homogeneous distribution of gas over the sensor surfaces, thereby reducing system response time and increasing measurement stability.

#### 2.2.3. System Cleaning

**Figure 4:**
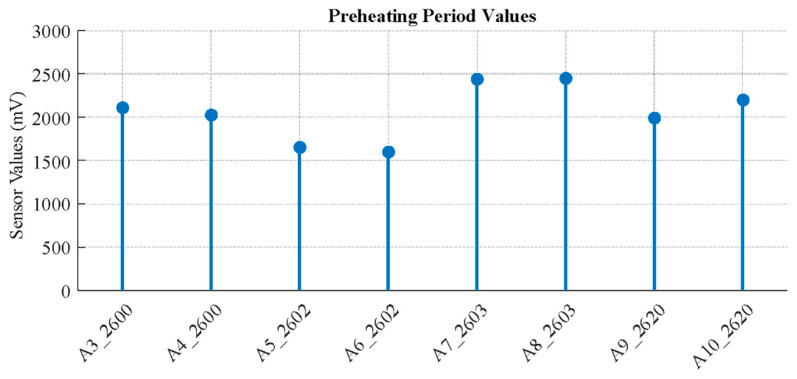
Sensor data after 30-min preheating period.

**Figure 5:**
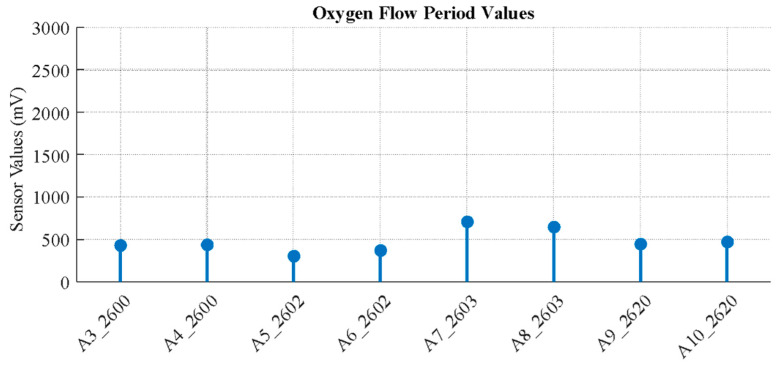
Sensor data after 2-min oxygen flow.

A cleaning procedure is applied before each experiment by supplying pure oxygen to the system. Pure oxygen flow successfully resets the sensors by gradually driving their readings towards a “0” value, which was confirmed to be a suitable and non-damaging cleaning method (Figure 4 and Figure 5).

All experiments are conducted under controlled laboratory conditions at an ambient temperature of approximately 23–25 °C. Prior to each measurement session, the electronic nose system is allowed to stabilize through a 30-min preheating period to ensure consistent baseline signals and sensor response stability.

### 2.3. Olfactometry Method Integration

The TS EN 13725 standard defines one odor unit (1 OU/m^3^) as the mass of odorant equivalent to 1.659 µmol of n-butanol per 1 m^3^ of neutral gas, which is evaluated by a trained panel. This standard was used to obtain human perception data.

#### 2.3.1. Panelist N-Butanol Calibration

Panelist selection and calibration rely on determining the odor detection threshold ($Z_{ITE}$) using n-butanol gas diluted at various concentrations. The individual detection threshold ($Z_{ITE,i}$) is calculated based on the geometric mean of the transition region dilutions from “yes/no” responses. The group detection threshold ($\bar{Z_{ITE}}$) for n panelists is:

(1) $$\bar{Z_{ITE,i}}={\left(\prod_{i=1}^{n}Z_{ITE,i}\right)}^{\frac{1}{n}}$$

Panelists are considered “Expert Noses” if their detection thresholds fall within the 20–80 ppb range [1].

#### 2.3.2. Odor Concentration Measurement

The odor concentration ($C_{odor}$) of a sample gas is determined by the geometric mean of the individual detection thresholds ($Z_{ITE}$) obtained from the calibrated Expert Nose panel:

(2) $$C_{odor}=\bar{Z_{ITE}}\times 1\;{OU}_{E}/m^{3}=\bar{Z_{ITE}}\;{OU}_{E}/m^{3}$$

**Figure 6:**
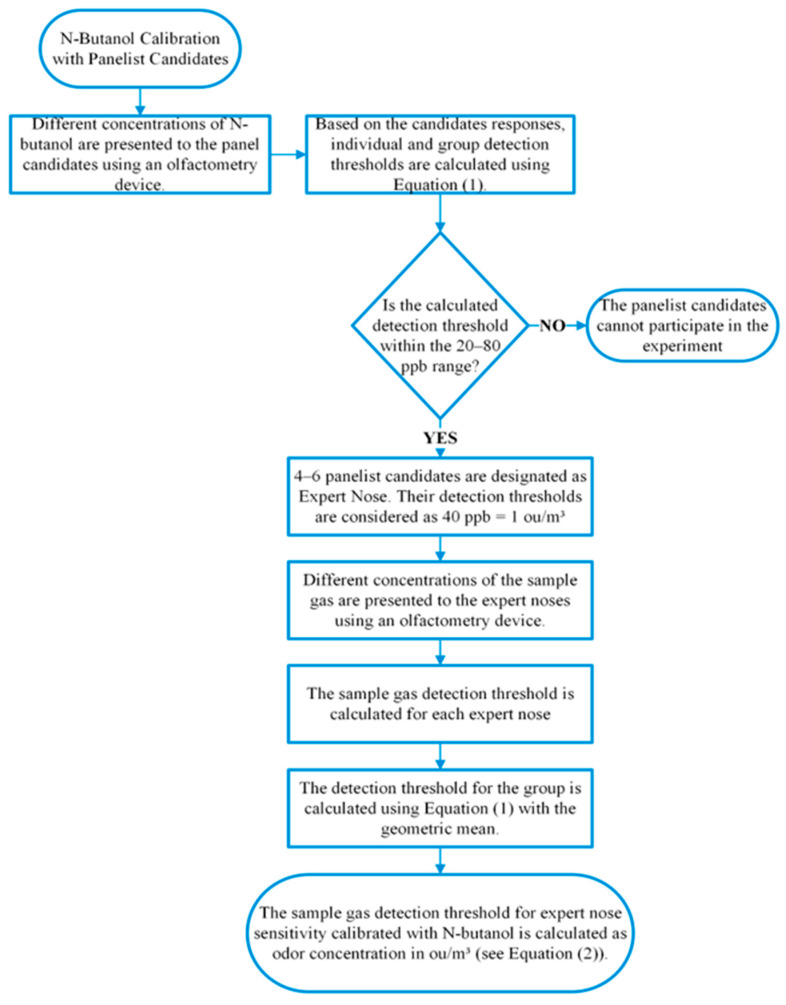
Workflow of the olfactometry method.

The workflow for this method is summarized in Figure 6.

According to the TS EN 13725 standard, panelists are calibrated using n-butanol gas with detection thresholds typically ranging between approximately 20–80 ppb, providing readers with a sense of the odor concentration. Odor units (OU/m^3^, referred to as OU_E_/m^3^ in the standard) remain a perception-based metric and cannot be directly converted to ppm or ppb for complex odor mixtures.

Human data are obtained by asking participants to indicate whether they can perceive the odor (“Yes” or “No”). Table 2 provides an example of the olfactometry panelist data used for threshold determination.

Following these standard calibration procedures, a pungent onion extract was selected as a complex test material to evaluate the system’s practical performance. This specific target was chosen because its multi-component volatile profile allowed us to concurrently record the electronic nose responses and precisely correlate them with the comprehensive olfactometry data obtained from the panelists. To create an onion sample with a known odor value, water was first removed from the onion, and deionized water was added to create a 1/512 concentration stock solution. Stepwise dilutions from this stock are then prepared at 1/1024, 1/2048, 1/4096, and 1/8192 concentrations. Experimental measurements show that the 1/512 and 1/1024 concentrations cause sensor saturation, preventing reliable data collection. In contrast, sensor responses at the 1/2048 and 1/8192 concentrations are recorded reliably. To effectively process these reliable measurements, the sensor signals are continuously acquired via an STM32 Nucleo-F756ZG microcontroller board, enabling real-time monitoring through STM Studio v3.6.0 (STMicroelectronics, Geneva, Switzerland). Ultimately, these digitized multidimensional sensor responses are fed into the regression models developed within the MATLAB R2023b (The MathWorks, Inc., Natick, MA, USA) environment to execute the odor quantification pipeline.

### 2.4. Machine Learning Algorithms and Training/Testing Process

#### 2.4.1. Training Process

**Figure 7:**
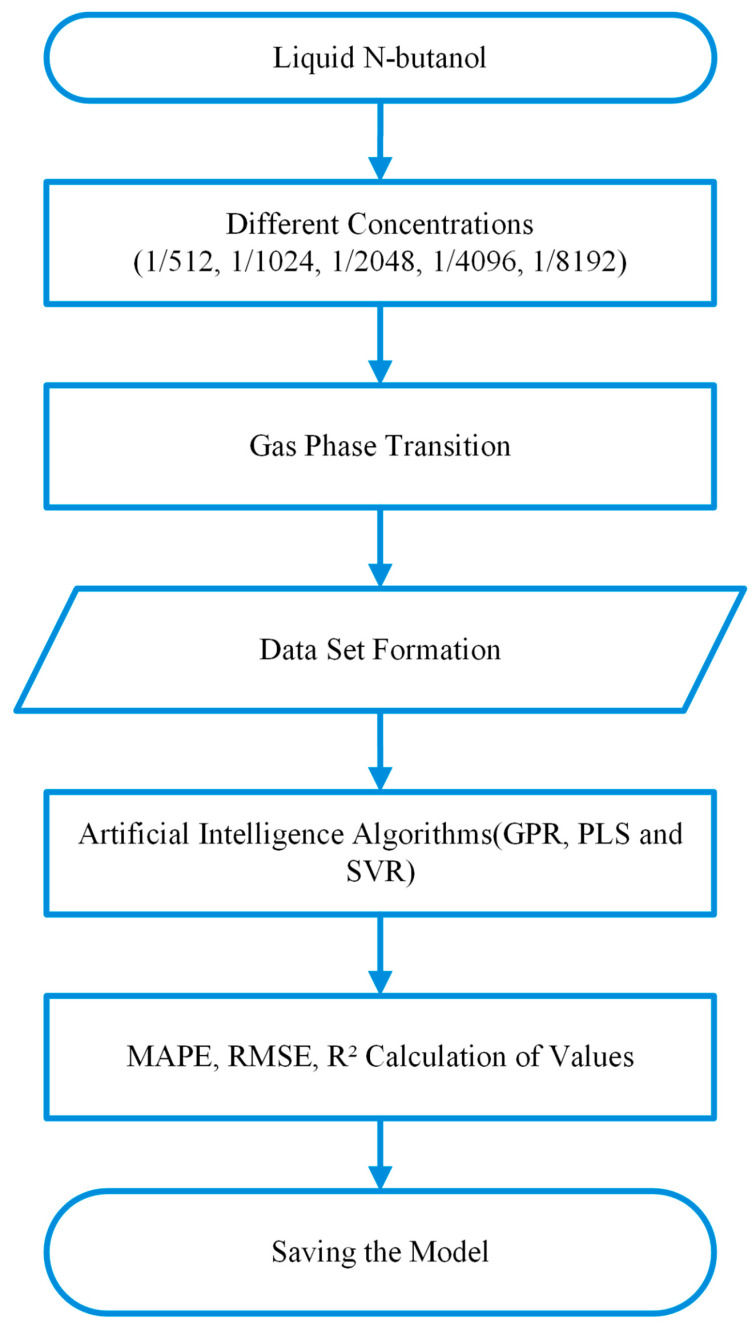
Training process flow diagram with n-butanol.

Following the data acquisition phase, the sensor responses to n-butanol samples (500 data points from eight sensors per concentration) were systematically recorded to create the training dataset. This dataset establishes the fundamental mapping between the multidimensional raw sensor signals and the known odor concentration (OU/m^3^) obtained from the olfactometry calibration. The entire pipeline, from data collection to model deployment, is summarized in the training process flow diagram presented in Figure 7. To enable robust multidimensional modeling of the obtained data from different perspectives and to evaluate comparative prediction capabilities, the dataset was fed into three distinct machine learning architectures:

- Gaussian Process Regression (GPR): Employed to capture complex nonlinear relationships effectively by modeling a probabilistic distribution over functions, thereby providing predictive uncertainty along with the regression estimates [28].
- Partial Least Squares (PLS): Utilized to represent highly correlated predictor variables with a small number of latent components that capture the underlying linear relationship with the response, enabling efficient dimensionality reduction and prediction [29].
- Support Vector Regression (SVR): Implemented as a robust supervised learning method that handles nonlinear patterns in high-dimensional spaces using kernel functions, maximizing the margin between predicted values and observed data to achieve strong generalization performance [30].

#### 2.4.2. Testing Process

**Figure 8:**
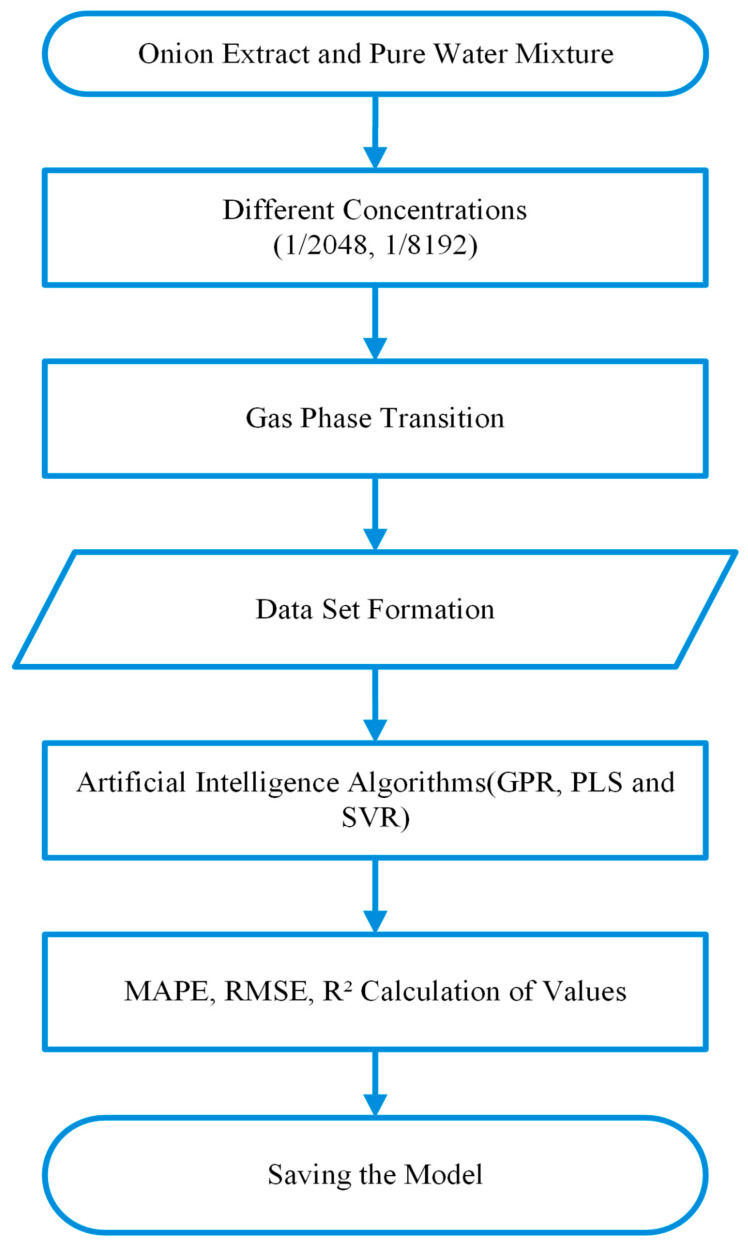
Onion data flow diagram.

The model training and optimization process was evaluated using standard statistical metrics: Mean Absolute Percentage Error (MAPE), Root Mean Square Error (RMSE), and the Coefficient of Determination (R^2^). The modeling involved two distinct evaluation stages to assess both learning fidelity and generalization capability. Initially, the n-butanol data—the primary reference substance—was used to calculate the Train MAPE, Train R^2^ and Train RMSE. These metrics quantified the models’ learning success and their ability to fit the patterns within the data they were trained on. The model achieving the highest accuracy with the n-butanol training data was then integrated into the system for real-world testing. The holistic testing process, from experimental data collection to artificial intelligence model evaluation, is summarized in Figure 8.

In the subsequent testing phase, onion samples were used as an independent dataset to compute the Test MAPE, Test R^2^ and Test RMSE. This independent onion data was introduced to assess the model’s generalization success, demonstrating its performance and reliability when predicting odor values for a chemical profile significantly different from the n-butanol compound used during the training phase. Onion samples were strategically used as the test material, as their complex profile containing sharp, volatile compounds provides a necessary contrast to the single-component n-butanol reference. Furthermore, onion samples were diluted in a standardized manner (at 1/2048 and 1/8192 concentrations) to allow comparison on the OU/m^3^ scale, ensuring the measurements fell within the sensor’s functional range.

## 3. Results

### 3.1. Machine Learning Model Performance

The performance of the PLS, SVR, and GPR models on the n-butanol training dataset and the independent onion test dataset is compared in Table 3.

### 3.2. Performance Analysis

The numerical results highlight the characteristic features of each model:

- PLS Model: Demonstrated fast and stable modeling with excellent fit on the training set (R^2^: 0.997). Its strong Test R^2^ (0.957) suggests good generalization and computational efficiency for high-dimensional data.
- SVR Model: Showed the best generalization performance on the test set. Despite a slightly higher Training MAPE, the significant reduction in Test RMSE (0.056) and the highest Test R^2^ (0.987) underscore its superior capability in flexibly modeling complex, nonlinear data relationships.
- GPR Model: Exhibited a near-perfect fit on the training data (R^2^: 1.000, MAPE: 0.148%). However, its reduced success on the test set suggests a degree of overfitting to the training data. Nonetheless, the high test R^2^ (0.935) confirms its strong explanatory power.

While numerous electronic nose studies in the literature, such as those evaluating fish freshness using a Decision Tree Model [6] or achieving near 100% distinguishability in beverage analysis [5], focus on classification problems (determining which category a sample belongs to), the proposed method addresses a more complex regression problem. This method’s objective is to precisely quantify the odor concentration based on the TS EN 13725 standard, correlating sensor signals with a numerical, standardized value derived from olfactometry panel evaluations. Although the literature often cites high classification accuracies (e.g., 90.7% or near 100%), which gauge the success of assigning a sample to the correct category, our metrics are fundamentally different. The superior performance of our SVR model, evidenced by the highest Test R^2^ of 0.987 and a low Test RMSE of 0.056, signifies its capability not merely to categorize the odor, but to objectively and accurately predict a standardized concentration value. This prediction capability confirms that the proposed methodology directly addresses the critical objective in environmental engineering: developing an objective, standardized, and reproducible instrumental alternative to subjective human-perception-based measurements.

### 3.3. Concentration-Dependent Prediction

This section illustrates the prediction performance at different concentration ranges, comparing the predicted odor values with the actual olfactometry measurements. It should be noted that measurements at each concentration level were performed sequentially within a continuous experimental session, with oxygen cleaning applied between measurements to restore baseline conditions. However, due to the inherent dynamic behavior of MOX sensors, including recovery limitations, thermal stabilization, and surface adsorption effects, the sensor response may still exhibit time-dependent variations. In addition, standard preprocessing steps were applied to reduce the influence of anomalous readings. Consequently, smooth trends may appear in the data even under constant concentration conditions.

#### 3.3.1. Low Concentration

**Figure 9:**
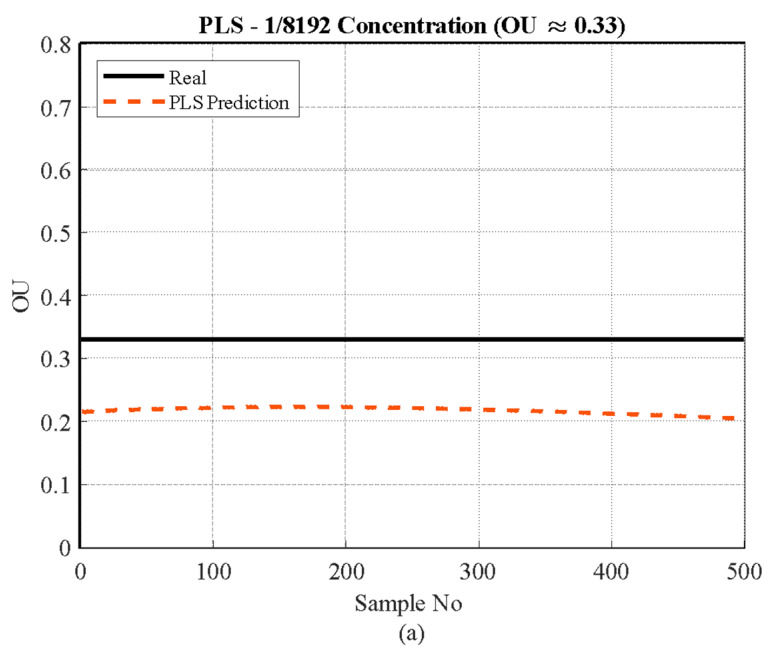

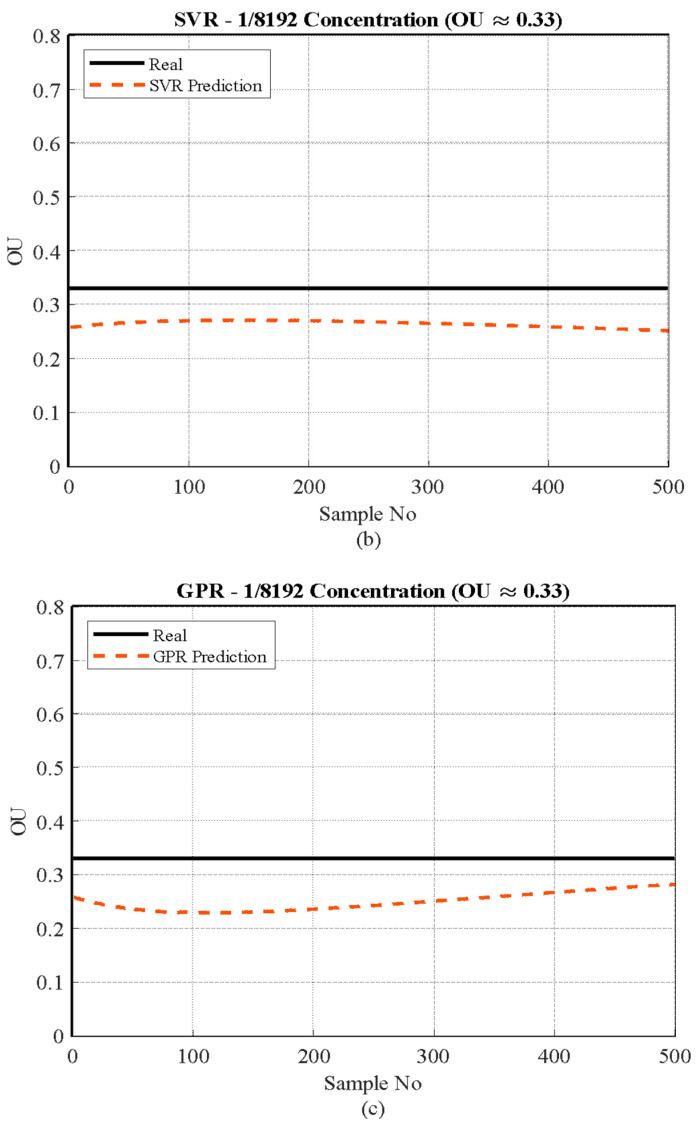
Actual vs. predicted values for the 1/8192 concentration range (≈0.33 OU/m^3^) using three regression models: (**a**) PLS, (**b**) SVR, and (**c**) GPR.

Figure 9 illustrates the overall prediction performance of the models for n-butanol at a low concentration level (approximately 0.33 OU or 1/8192 dilution). A consistent, yet minor, systematic deviation is observed between the predicted and true values across all models at this detection threshold, highlighting the challenge of quantifying odors near the sensor arrays’ minimum effective range.

The Partial Least Squares (PLS) model (Figure 9a), consistent with its linear nature and advantages in handling correlated data, exhibits high stability across all 500 samples, maintaining a nearly flat prediction line. However, the model significantly underestimates the true value, stabilizing around 0.21 OU. The Support Vector Regression (SVR) model (Figure 9b), despite its capability to manage non-linear relationships via kernel functions, demonstrates a similar systematic underestimation, although its prediction line stabilizes slightly closer to the Real Value (around 0.25 OU), reflecting a minimal improvement in accuracy over PLS. Conversely, the Gaussian Process Regression (GPR) model (Figure 9c), which excels at probabilistic modeling and capturing complex non-linear dependencies, exhibits the best fit to the true value among the three tested models at this low threshold. While the GPR prediction still systematically underestimates the Real Value of 0.33 OU (reaching a maximum of approximately 0.28 OU), the magnitude of this error is the smallest observed. This suggests that GPR’s inherent flexibility allows it to extract slightly more accurate information from the small and potentially noisy sensor signals near the system’s detection limit compared to the more constrained PLS and SVR models.

#### 3.3.2. High Concentration

**Figure 10:**
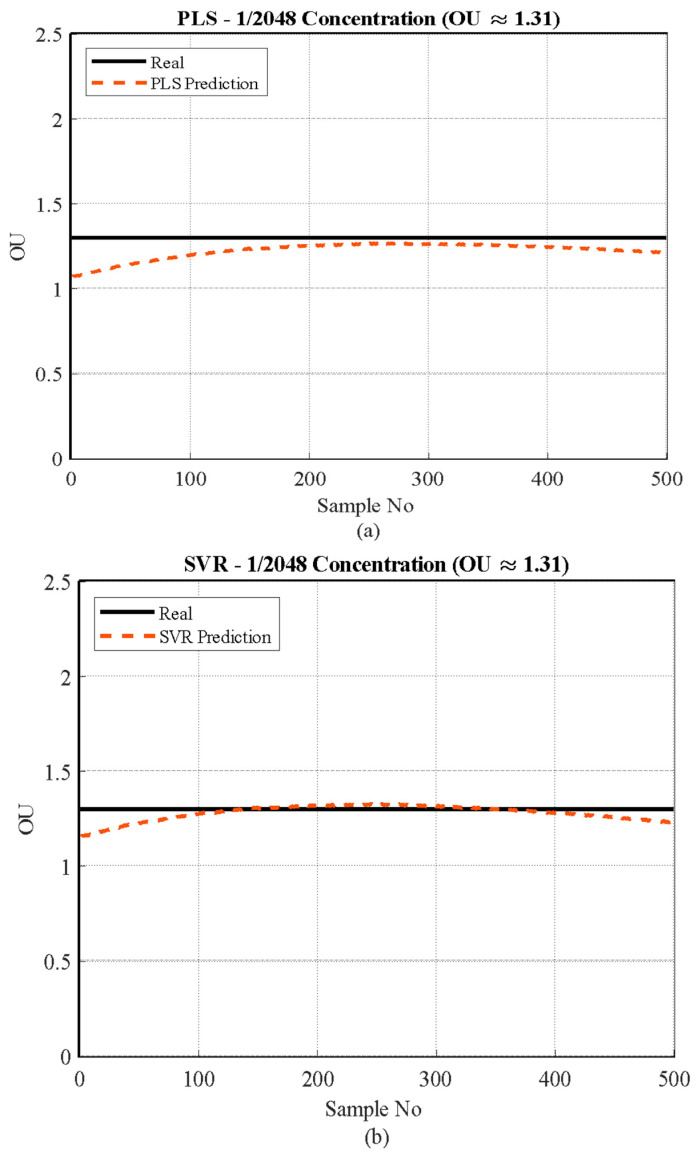

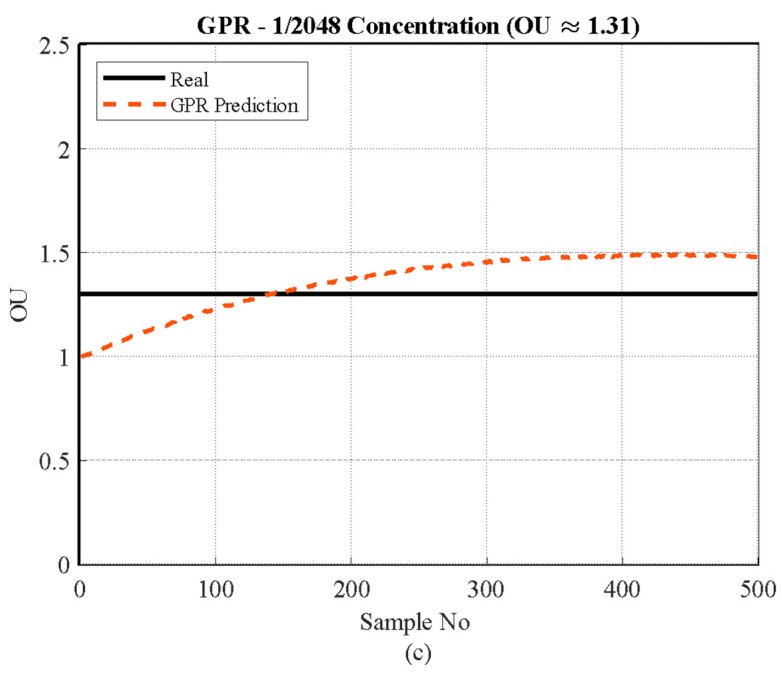
Actual vs. predicted values for the 1/2048 concentration range (≈1.31 OU/m^3^) using three different models: (**a**) PLS, (**b**) SVR, and (**c**) GPR.

Figure 10 illustrates the overall prediction performance of the three models for n-butanol at the medium-high concentration level of 1/2048 (approximately 1.31 OU). This higher signal range reveals distinct generalization capabilities among the algorithms, unlike the systematic underestimation observed at the low detection limit.

The Support Vector Regression (SVR) model (Figure 10b) demonstrated clear superiority in this range, rapidly converging and achieving an almost perfect match with the Real Value of 1.31 OU, confirming its effectiveness in modeling complex non-linear sensor data at optimal operating points where the signal-to-noise ratio is high. In contrast, the Partial Least Squares (PLS) model (Figure 10a), although exhibiting high stability across all 500 samples, consistently underestimated the true value, stabilizing just below 1.31 OU. This confirms that the inherent linear constraint of the PLS model prevents it from perfectly capturing the mapping function, resulting in a residual error even when stable, high-magnitude signals are provided. Conversely, the Gaussian Process Regression (GPR) model (Figure 10c) showed the least accurate performance in this range by systematically overestimating the true value, stabilizing near 1.48 OU. This pronounced divergence highlights GPR’s tendency toward over-sensitivity; despite its strong probabilistic performance at the low detection limit, its high flexibility leads to poor generalization and significantly higher error when extrapolating to the stable, higher-magnitude signals of the medium-high concentration range.

## 4. Discussion

This study successfully established a robust electronic nose (E-nose) system framework for objective odor quantification, validating its performance directly against the internationally recognized TS EN 13725 olfactometry standard using n-butanol as the primary reference. This approach met the core research objective by confirming a strong correlation between the e-nose sensor responses and the panelist-derived odor unit (OU/m^3^) values, thus demonstrating that the system can minimize the inherent subjectivity and logistical burden associated with human sensory measurements.

The quantitative assessment of odors using sensor-based systems is inherently influenced by the sample matrix, as volatile compound release varies significantly depending on composition (e.g., water, lipids, or polymers). In this study, the system was fundamentally calibrated using n-butanol in accordance with the TS EN 13725 standard, ensuring a standardized baseline for chemical odor quantification. The onion extract was intentionally introduced only during the testing phase as a complex and multi-component matrix to evaluate the generalization capability of the proposed AI model.

This design also reflects a major challenge in food aroma analysis, where matrix complexity and background interferences significantly affect sensor responses and odor perception. The ability of the proposed system to generalize from a pure reference compound to a complex matrix demonstrates its robustness. Future applications of the device include not only food aroma intensity assessment but also environmental monitoring and the detection of hazardous chemical compounds.

The analysis of machine learning models—PLS, SVR, and GPR—to interpret the multidimensional sensor data revealed distinct performance characteristics across different concentration ranges. Crucially, the Support Vector Regression (SVR) model demonstrated superior generalization capability on the independent onion odor dataset, achieving the highest Test R^2^ (0.987) and the lowest Test MAPE of only 11.098%. In contrast, the PLS model yielded a Test MAPE of 19.823% while the highly flexible GPR model showed a relatively high Test MAPE of 17.556%. This achievement is particularly significant when compared to most existing E-nose studies in the literature (e.g., [5,6,12]) which typically report high classification accuracies (e.g., 90–100%) for distinguishing odor categories. Unlike those studies, this work successfully performs a more challenging quantitative task (regression), accurately predicting the concrete OU/m^3^ value itself. This quantitative success, coupled with the TS EN 13725 validation, lends unprecedented regulatory relevance and reliability to the E-nose measurements, confirming SVR as the most reliable and flexible algorithm for this framework. To fully assess the practical prospects and advantages of the proposed AI-enabled electronic nose, it is essential to compare it with established analytical methods and widely used commercial hardware solutions in terms of analysis time, price, and required operator qualifications (Table 4).

Advanced analytical platforms, such as HS-SPME-GC-MS, provide precise chemical characterization of individual volatile compounds [18]. However, they suffer from very high equipment costs, lengthy analysis times due to complex sample equilibration, and require highly trained analytical chemists. On the other hand, widely used commercial electronic noses, such as the i-Pen/PEN3 (Airsense Analytics GmbH, Schwerin, Germany) systems or the Cyranose 320 (Sensigent, Baldwin Park, CA, USA), offer rapid results for environmental monitoring and chemical classification. Nevertheless, these traditional e-noses typically aim for specific chemical characterization or relative detection using large sensor arrays, often without directly correlating their output to a universally standardized and legally binding odor metric.

The system proposed in this study distinguishes itself by operating on the principle of generalization rather than chemical characterization. By leveraging the SVR model, the system successfully translates multi-dimensional sensor signals from a cost-effective, broad-spectrum Metal Oxide (MOX) array directly into standardized OU/m^3^ values. Because the AI model generalizes the complex sensor responses into a single intensity metric, it does not require an extensive variety of highly specific sensors. This approach drastically reduces the overall hardware price, eliminates the need for complex sample preparation, and minimizes operator qualification requirements.

Additionally, the E-nose system offers clear advantages over traditional dynamic olfactometry, which inherently relies on trained human panels and accredited laboratories to produce standardized odor measurements. Human-based olfactometry requires extensive preparation, scheduling of multiple expert assessors, and continuous verification of sensory thresholds, all of which contribute to high operational costs, long analysis times, and limited reproducibility. In contrast, our AI-enabled E-nose delivers rapid, objective, and reproducible measurements without reliance on human perception. Its ability to provide quantitative odor data in standardized units (OU/m^3^) with high consistency across repeated measurements represents the key innovation of this system. These distinguishing features underscore the practical applicability of the E-nose for environmental monitoring, industrial process verification, and field-deployable odor assessment, effectively bridging the gap between laboratory-based human assessment and automated, real-world odor quantification.

In conclusion, this research provides a significant methodological contribution by systematically assessing E-nose technology using a standardized reference. The reliability and speed demonstrated by the SVR-supported E-nose system strongly validate its potential as a low-cost, repeatable, and highly accurate alternative for critical real-world applications, particularly in environmental odor monitoring, food quality control, and industrial process verification. Future work should focus on expanding the model validation to include a wider array of complex gas mixtures and evaluating the system’s long-term stability in diverse field conditions.

## Figures and Tables

**Table 1:** Characteristics of FIGARO TGS26 Series Gas Sensors.

Sensor Code	Target Gas	Features	Range (ppm)
A3_TGS2600 A4_TGS2600	Air pollutants	High sensitivity	Hydrogen: 1–30
A5_TGS2602 A6_TGS2602	Air pollutants	High sensitivity to VOCs, H_2_S, and ammonia	Ethanol: 1–30
A7_TGS2603 A8_TGS2603	Air pollutants	High sensitivity to amines and sulfur-containing odors	Ethanol: 1–30
A9_TGS2620 A10_TGS2620	Alcohols	High sensitivity to organic solvent vapors	50–5000

**Table 2:** Olfactometry method results.

Analysis Parameter	Panelist	Odor Unit (OU/m^3^)
n-Butanol	Participant 1	2047.87
	Participant 2	
	Participant 3	
	Participant 4	
	Participant 5	
	Participant 6	

**Table 3:** Artificial intelligence comparison table.

Model	Train MAPE (%)	Test MAPE (%)	Train RMSE	Test RMSE	Train R^2^	Test R^2^
PLS	6.534	19.823	0.384	0.101	0.997	0.957
SVR	10.891	11.098	1.709	0.056	0.95	0.987
GPR	0.148	17.556	0.015	0.124	1.000	0.935

**Table 4:** Comprehensive comparison of the proposed AI-enabled E-Nose with established odor and VOC assessment methods.

Feature	Dynamic Olfactometry (TS EN 13725)	Analytical Methods (e.g., GC-MS)	Commercial E-Noses (e.g., PEN3, Cyranose 320)	Proposed AI-Enabled E-Nose
Primary Objective	Odor Intensity (OU/m^3^)	Chemical Characterization	VOC Classification/ Relative Detection	Odor Intensity (OU/m^3^)
Analysis Time	High (Hours/Days)	High	Low (Minutes)	Low (Minutes)
Price/Cost	High (Recurrent human panel costs)	Very High (Capital & operational)	Medium to High (Application dependent)	Low (MOX sensors, low diversity needed)
Required Qualifications	High (Accredited lab, trained panel)	Very High (Analytical chemist)	Medium (Trained operator)	Low (Automated AI processing)
Standard Compliance	Yes (Gold Standard)	No	No	Yes (Correlated to TS EN 13725)
Hardware Complexity	Not Applicable (Human Nose)	Very High (Chromatographic columns, vacuum)	Medium to High (Large sensor arrays needed)	Low (Generalization via AI + base sensors)

## Data Availability

The data presented in this study are available on request from the corresponding author.
